# A Treatise on Amaurosis and Amaurotic Affections

**Published:** 1840-07

**Authors:** 


Art. V.—*A Treatise on Amaurosis and Amaurotic Affections.* By Edward Octavius Hocken.—*London*, 1840. 8vo, pp. 359.

This is a beautiful and elegantly printed volume, almost in itself sufficient to cure the Tweedican amaurosis, noticed in another article of this Number. We have had recourse to it for this very purpose, and with a very satisfactory result. But, great as they are, the external merits are not the only merits of Mr. Hocken's book. To be sure it contains nothing original, but it contains almost all that has been said on the subject by others. In this compilation the industry of the author is conspicuous, and, we are bound to add, his candour also, as he always takes care to own his obligations to the authors from whom he borrows.

